# Comparing penalization methods for linear models on large observational health data

**DOI:** 10.1093/jamia/ocae109

**Published:** 2024-05-20

**Authors:** Egill A Fridgeirsson, Ross Williams, Peter Rijnbeek, Marc A Suchard, Jenna M Reps

**Affiliations:** Department of Medical Informatics, Erasmus University Medical Center, 3015 GD Rotterdam, The Netherlands; Department of Medical Informatics, Erasmus University Medical Center, 3015 GD Rotterdam, The Netherlands; Department of Medical Informatics, Erasmus University Medical Center, 3015 GD Rotterdam, The Netherlands; Department of Biostatistics, University of California, Los Angeles, Los Angeles, CA 90095-1772, United States; VA Informatics and Computing Infrastructure, United States Department of Veterans Affairs, Salt Lake City, UT 84148, United States; Department of Medical Informatics, Erasmus University Medical Center, 3015 GD Rotterdam, The Netherlands; Observational Health Data Analytics, Janssen Research and Development, Titusville, NJ 08560, United States

**Keywords:** logistic regression, electronic health records, regularization, discrimination, calibration

## Abstract

**Objective:**

This study evaluates regularization variants in logistic regression (L1, L2, ElasticNet, Adaptive L1, Adaptive ElasticNet, Broken adaptive ridge [BAR], and Iterative hard thresholding [IHT]) for discrimination and calibration performance, focusing on both internal and external validation.

**Materials and Methods:**

We use data from 5 US claims and electronic health record databases and develop models for various outcomes in a major depressive disorder patient population. We externally validate all models in the other databases. We use a train-test split of 75%/25% and evaluate performance with discrimination and calibration. Statistical analysis for difference in performance uses Friedman’s test and critical difference diagrams.

**Results:**

Of the 840 models we develop, L1 and ElasticNet emerge as superior in both internal and external discrimination, with a notable AUC difference. BAR and IHT show the best internal calibration, without a clear external calibration leader. ElasticNet typically has larger model sizes than L1. Methods like IHT and BAR, while slightly less discriminative, significantly reduce model complexity.

**Conclusion:**

L1 and ElasticNet offer the best discriminative performance in logistic regression for healthcare predictions, maintaining robustness across validations. For simpler, more interpretable models, L0-based methods (IHT and BAR) are advantageous, providing greater parsimony and calibration with fewer features. This study aids in selecting suitable regularization techniques for healthcare prediction models, balancing performance, complexity, and interpretability.

## Introduction

A recent review of the use of clinical prediction models finds that in recent years 67% of studies use some kind of regression analysis.^1^ One common issue when developing clinical prediction models is the susceptibility to overfitting. Overfitting occurs when a model is overly complex such that it near perfectly fits the training data but is unable to generalize to new data. A common method to reduce overfitting when training regression models involves adding a regularization term to the model’s objective function. The regularization term adds a cost that aims to minimize model complexity. This is known as regularization. Studies on large observational health data commonly use L1 regularized logistic regression, otherwise known as Least Absolute Shrinkage and Selection Operator (LASSO) for its feature selection capabilities and good discriminative performance.^2^ As machine learning methods are advancing, pipelines to develop clinical prediction models, that work directly on large observational healthcare data (insurance claims and electronic healthcare records), are being developed. These pipelines develop models efficiently and improve model transparency. A recent study highlighting the use of a standardized analytical pipeline on observational health-data mapped to the Observational Health Data Sciences and Informatics (OHDSI) common data model (CDM) shows that logistic regression with LASSO often outperforms other machine learning models when externally validating the developed model.^3^ External validation is when the model is validated on a different dataset than it was trained on. It is common for developed models to have a drop in performance when transported to a different dataset and one of the outstanding issues in developing prediction models on observational data is to develop more generalizable models which do not suffer from this drop.^4^

LASSO regularization is a method developed at the end of the last century. It has enjoyed great success due to its interpretability and scalability^5^ and has been heavily used since its inception. Despite its success it has some limitations, especially regarding its feature selection capabilities. These limitations have led researchers to develop numerous other variants with differing theoretical properties. One such limitation is that if there is a group of features in the data with high pairwise correlations within the group, LASSO tends to select randomly one of the features as a representative of the whole group and push the rest to zero.^6^ Correlations between features are common when using standardized analytical pipelines in healthcare due to comorbidities and coding redundancies. Another limitation is that the LASSO feature selection is unstable unless specific conditions are met which rarely happens in practice.^7^ This means that the covariates selected will change drastically with minor changes in the data distribution. In practice this means that fitting models with LASSO regularization for example on different folds during cross validation can result in models selecting different features. Further there is an issue of false positives among the selected features. While LASSO is highly likely to select the relevant features, this is at the cost of features that are not relevant but end up in the model.^8^ This is worse when there is high collinearity among the features as is common in observational health data.^9^

To address these issues several variants of the LASSO have been developed. LASSO regularization incorporates an L1 penalty into a model’s objective function by considering the absolute value of the coefficients’ magnitude. This leads some coefficients to be pushed to precisely zero selecting some features while discarding others. L2 regularization on the other hand, known as Ridge,^10^ introduces a penalty based on the squared magnitude of the coefficients. Unlike L1, L2 does not force any coefficient to become exactly zero and all features are included in the final model. One of the first variants introduced to improve the LASSO was ElasticNet^6^ which use a mixture of L1 and L2 penalty. This means that in the presence of groups of correlated features it will select the whole group while still maintaining its feature selection capacity. ElasticNet has been shown to be more stable in feature selection than LASSO.^11^ Another developed variant is the adaptive LASSO^12^ where each feature is adaptively penalized according to an estimation of its coefficient magnitude. This means larger coefficients are penalized less, which reduces bias, and smaller coefficients are penalized more which results in sparser (ie, selects less features) and more stable models where fewer false positive features are selected.^13^^,^^14^ A more recent adaptive variant is the broken adaptive ridge (BAR).^15^^,^^16^ This is a version where ridge regression is iteratively fitted and in each iteration the coefficients are penalized according to the inverse of the magnitude of their coefficient in the previous iteration, this is repeated until convergence. This variant has the desired property that it is an approximation for L0 regularization, which is equal to best subset selection where every possible combination of features is tried to select the best subset. Finally, another L0 approximation method is often used; iterative hard thresholding (IHT).^17^ IHT has the advantage that a maximum number of selected features can be specified which can be attractive in a clinical setting.

In this study, we empirically examine these different regularization variants and investigate their discrimination and calibration performance both when evaluated on the same dataset as they are developed on (internal validation) and on a different dataset (external validation) and compare to the LASSO.

## Methods

### Data source

We use 5 US claims and electronic health record (EHR) databases (Table 1). We develop our models on 1 database and externally validate on the other 4, then repeat for all databases as the development database. The 5 databases in this study contain retrospectively collected deidentified patient healthcare data. The use of IBM and Optum databases were reviewed by the New England Institutional Review Board (IRB) and were determined to be exempt from broad IRB approval.

**Table 1.** Overview of data sources used in the study.

Name	Type	Description	Start	End	Size (million lives)
IBM^®^ MarketScan^®^ Commercial Claims and Encounters (CCAE)	US Claims	Patients aged 65 or younger. Employees who receive health insurance through their employer and their dependents	January 01, 2000	July 31, 2021	152
IBM^®^ MarketScan^®^ Medicare Supplemental (MDCR)	US Claims	Patients aged 65 or older with supplemental healthcare	January 01, 2000	July 31, 2021	10
IBM^®^ MarketScan^®^ Medicaid (MDCD)	US Claims	Patients with government subsidized healthcare	January 01, 2006	May 31, 2021	33
Optum^®^ de-identified Electronic Health Record Dataset (Optum EHR)	US EHR	Patients of all ages	January 01, 2007	December 31, 2021	106
Optum^®^ De-Identified Clinformatics^®^ Data Mart Database (Clinformatics^®^)	US Claims	Patients of all ages	May 01, 2000	March 31, 2022	93

All datasets used in this paper were mapped into the OHDSI Observational Medical Outcomes Partnership Common Data Model (OMOP-CDM) version 5.^18^ The OMOP-CDM was developed to enable researchers with diverse datasets to have a standard database structure and vocabulary. This enables analysis code and software to be shared among researchers and across data sources which facilitates external validation of prediction models.

### Study population

**Figure 1.**
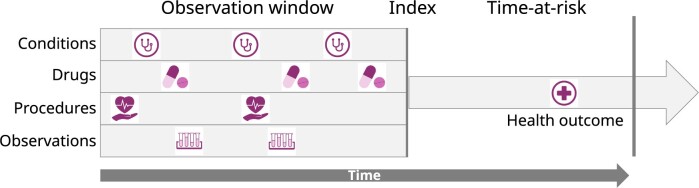
A patient level prediction problem. Conditions, drugs, procedures, and observations from an observation window prior to an index date are used to predict the outcome during a time-at-risk after index. Reproduced from John et al^19^ with permission from *BMC Medical Research Methodology*.

The target population consists of patients with pharmaceutically treated major depressive disorder (MDD) and the index date is their first diagnosis of MDD. We use individual patient features from an observation window of 1 year prior to index, and attempt to predict 21 different outcomes from 1 day to 1 year after the index date (See Figure 1). These are the same prediction problems as have been used previously.^20^ The outcomes are acute liver injury, acute myocardial infarction, alopecia, constipation, decreased libido, delirium, diarrhea, fracture, gastrointestinal hemorrhage, hyponatremia, hypotension, hypothyroidism, insomnia, nausea, seizure, stroke, sudden cardiac death, suicide and suicidal ideation, tinnitus, ventricular arrhythmia, and vertigo.

### Features

The individual patient features included are continuous age at index, sex, conditions, drug ingredients, procedures, and observations. These are all extracted from an observation window of 1 year prior to the index date. Conditions, drug ingredients, procedures, and observations are extracted as binary indicator variables with the value 1 indicating the presence of the feature in the observation window. In the context of the OMOP-CDM, conditions indicate the presence of a disease, sign or symptom, either observed by the healthcare provider or reported by the patient. Drug codes indicate exposures to drugs and are combined under the active ingredient to reduce feature dimensionality. Procedures are activities or processes carried out by the healthcare provider and observations are clinical facts about a patient not captured by other clinical tables in the CDM.

As the features represent whether a patient has a condition/drug/procedure/observation record in the year prior to their prediction index, the values are never missing (0 = no record, 1 = record present). However, we do not impute missing records in the observational dataset, so in theory a patient may have a value of 0 for the feature “diabetes condition record in the prior year” but have diabetes unrecorded. Age is normalized by its max value in the training set and features rarer than 0.1% of observations are removed.

Further details about software used to extract features are in Supplementary Material.

### Algorithms

We use 7 different prediction algorithms to develop our models. They are all integrated into the PatientLevelPrediction (version 5.4.5) R package (https://github.com/OHDSI/PatientLevelPrediction/) to facilitate ease of development and external validation on data from the OMOP-CDM. The following algorithms are used to develop models: LASSO,^2^ L2 penalized logistic regression (Ridge),^10^ L1/L2 penalized logistic regression (Elastic Net),^6^ adaptive L1 regularized logistic regression (Adaptive LASSO),^12^ adaptive L1/L2 penalized logistic regression (Adaptive ElasticNet), BAR,^15^ and IHT.^17^ Further details about software used for model development are in the Supplementary material.

We develop models using a train-test split of 75%/25%. Most algorithms use a 3-fold cross validation^21^ on the training set in combination with grid search to select the best hyperparameters, except for BAR-BIC and IHT-BIC which use the Bayesian information criteria (BIC). Algorithms that use Cyclops for model fitting (see Supplementary material) uses auto search instead of grid search to find the best hyperparameter.^5^ For all these algorithms the hyperparameters are the strength of the penalty. We also include a version of BAR that uses 3-fold cross validation and a simple grid search of 10 penalties, ranging from BIC to 0.1 * BIC, to select the best penalty based on the area-under-the-receiver-operating-characteristic curve (AUC). BAR and IHT require an initial fit of ridge regression which uses cross validation to find the optimal ridge regression penalty on the training set before refitting on the whole training set to get the initial regression coefficient estimates. Finally, all models are refit on the whole training set with fixed penalties set to the value from the hyperparameter search before internal validity is assessed on the test set. Since logistic regression models on this kind of data do not improve performance above around 3000 outcome events,^18^ if the training set includes more than 3000 events with a 75%-25% split the split was adjusted so the training set would only include 3000 events and the rest of the data would go to the test set to increase power in our performance estimates.

We use the AUC and the area under the precision-recall curve (AUPRC) to evaluate discriminative performance. The AUC is the most widely used and general metric to measure discrimination and does not depend on selecting a prediction threshold.^22^ AUPRC is another commonly used discrimination metric, particularly for rare outcomes.^23^ We evaluate calibration by computing the expected calibration error (ECE), also known as the E-avg.^24^ ECE measures the average difference of predicted versus expected risk in 10 equally spaced risk strata. We chose the ECE since we need a single metric for calibration that uses the whole range of risks. We develop the models on each database and then externally validate their performance on the other databases.

### Statistical analysis

We use Friedman’s test to compare the performance of different algorithms.^25^ This is a non-parametric test that detects if the different algorithms are ranked differently to each other on the different prediction problems. If the null hypothesis is rejected for a difference in ranks between the algorithms, we proceed with a post-hoc test testing all pairwise differences controlling for multiplicity. We plot the results from the post-hoc test in a critical difference diagram which show the ranks of each algorithm with horizontal lines connecting algorithms that are not significantly different from each other (see Section 3.2.4 in Demsar^25^ for further details). We do this for discrimination and calibration. We then look at distributions of model sizes of each model and compare them to the achieved performance.

## Results

### Study population

Table 2 provides an overview of study population statistics across the 5 different data sources. The age distributions are quite different between databases. CCAE and MDCD have younger people while MDCR has older. Optum EHR and Clinformatics^®^ are more spread in age.

**Table 2.** Study population characteristics.

	CCAE^b^	MDCR^c^	MDCD^d^	Optum EHR^e^	Clinformatics^®^^f^
Study population	2 220 724	181 912	628 293	3 140 079	1 649 138
Age (mean ± std)	41.1 ± 14.9	74.9 ± 7.8	33.6 ± 17.0	47.6 ± 19.1	50.7 ± 20.3
Sex (% male)	31.3%	32.8%	27.4%	30.9%	32.5%
No. of events^a^ (median, first, and third quartile)	12 358 (4619-27 575)	2363 (1736-5030)	5584 (2798-12 291)	17 624 (7627-46 724)	14 257 (8944-39 265)
Candidate predictors	42 219	29 548	36 697	72 254	47 254

a We have 21 different prediction problems per database, each with different number of outcome events.

b IBM® MarketScan® Commercial Claims and Encounter.

c IBM® MarketScan® Medicare Supplemental.

d IBM® MarketScan® Medicaid.

e Optum® de-identified Electronic Health Record Dataset.

f Optum® De-Identified Clinformatics® Data Mart Database.

### Discrimination

**Figure 2.**
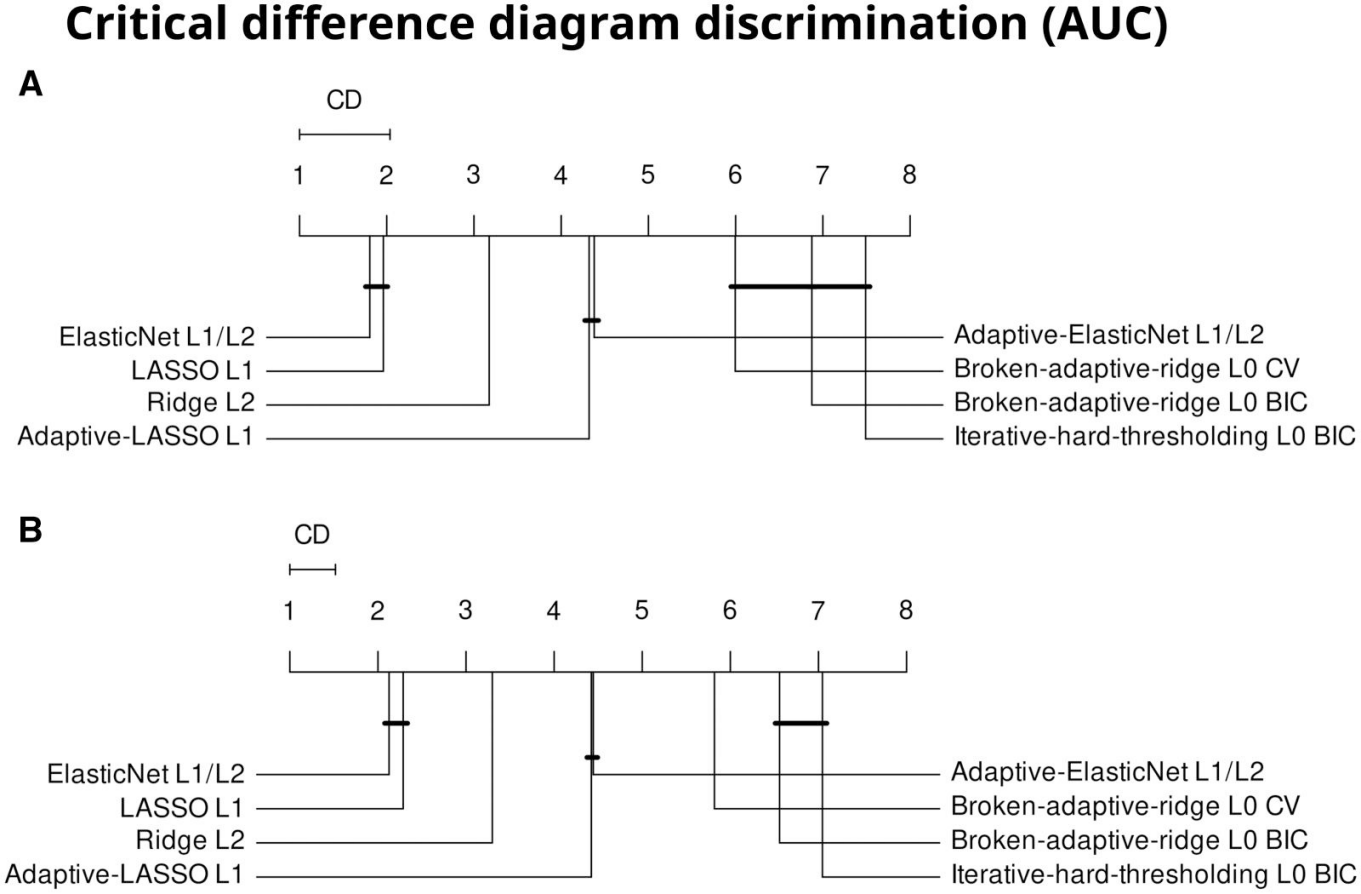
(A) Critical difference diagram of the developed models ranked using internal AUC. (B) Critical difference diagram ranked using external AUC. The critical difference (CD) line indicates how big of a difference is needed to be significantly different. Solid lines connect algorithms with no significant difference between them. Abbreviations: BIC = Bayesian information criteria, CV = cross validation.

In total we develop 840 models across the 5 databases (7 different algorithms with 2 hyperparameter tuning strategies for 1; predicting 21 outcomes; in 5 databases: 8 × 21 × 5 = 840). The AUC on the test set between the different algorithms are significantly differently ranked (Q(7) = 555, *P* <.001). Figure 2A shows a critical difference diagram for the internal discrimination performance. The best ranked algorithms are the LASSO and ElasticNet with no statistically significant difference between their ranks. Ridge is in third place, then the adaptive LASSO and adaptive ElasticNet, then the L0-approximate methods, IHT, and BAR. The average AUC difference between the best and worst algorithm is 2.8% points. Practically this mean that when randomly selecting 1 person with and 1 without the outcome, the best algorithm will get the order correct (assign higher risk to person with outcome) on average 2.8% points more often than the worst algorithm. We then externally validate the 840 developed models across the other 4 databases resulting in 3360 external validations (840 models × 4 databases). We find that the external discrimination performance measured using AUCs is significantly different between the algorithms (Q(7) = 1678, *P* <.001). Figure 2B shows the critical difference diagram for the external discrimination performance. The ranks of algorithms are the same as during internal performance. LASSO and ElasticNet show the best performance while the approximate L0 methods rank last. The average difference between the best and worst algorithm is 2.8% points AUC. In Figure S1 results with the area under the precision-recall curve and in Tables S1 and S2 the ranks per each of the 21 outcomes are shown.

### Calibration

**Figure 3.**
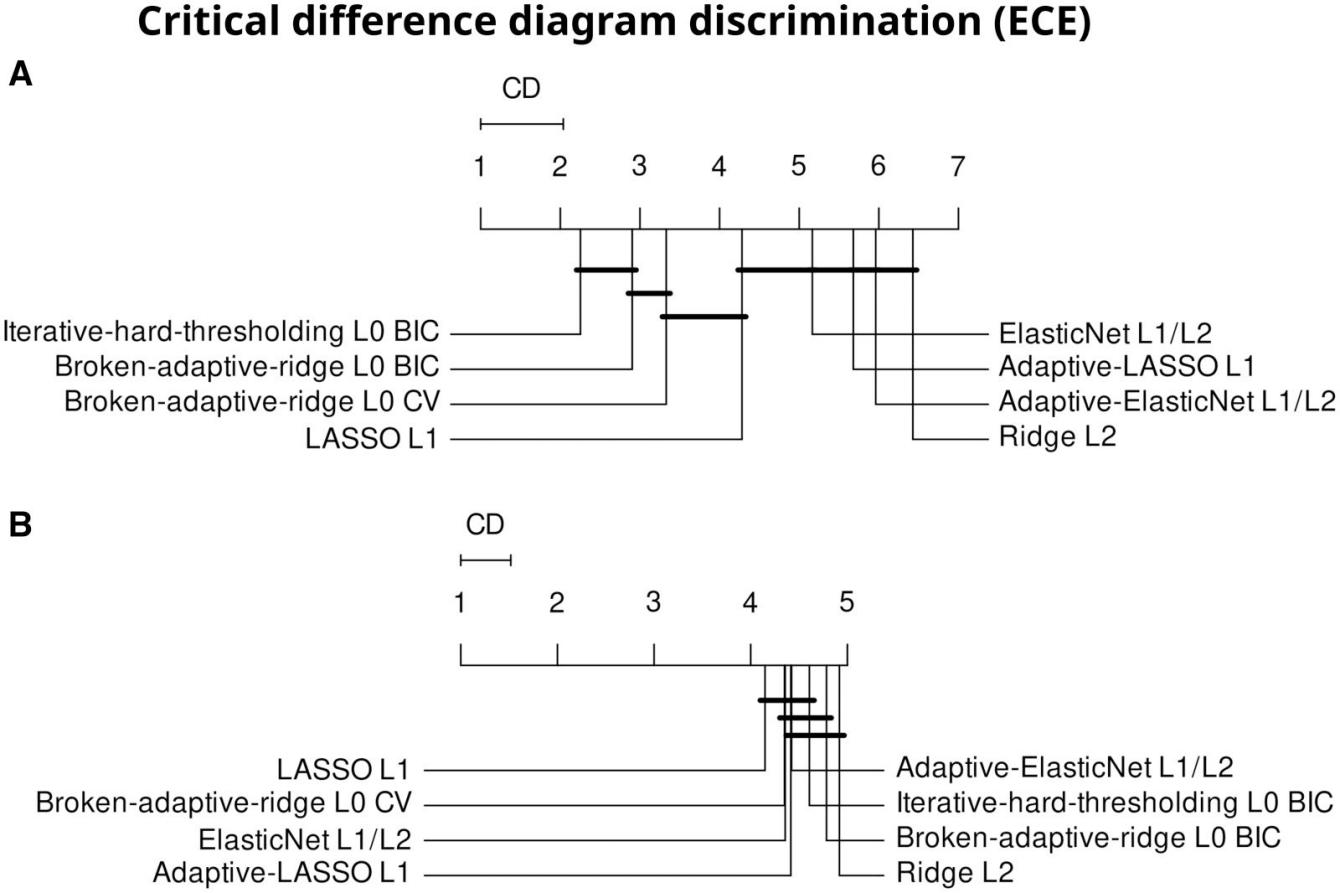
Expected calibration error (ECE) ranked according to (A) internal and (B) external performance. Abbreviations: CD = critical difference, BIC = Bayesian information criteria, CV = cross validation.

Figure 3 shows the results for the ECE. The algorithms are ranked differently for internal calibration performance (Q(7)=286.7, *P* <.001). The approximate L0 methods, BAR, and IHT lead in calibration. The adaptive LASSO, adaptive ElasticNet, and Ridge regression rank worst. The magnitude of difference between the best and worst algorithm is small or only 0.001. Externally the algorithms rank differently (Q(7)=31, *P* <.001). However, there is more variation in the ranks, with all average ranks between 4 and 5, and the magnitude of difference between the best and worst algorithm is small or less than 0.001.

### Model sizes

**Figure 4.**
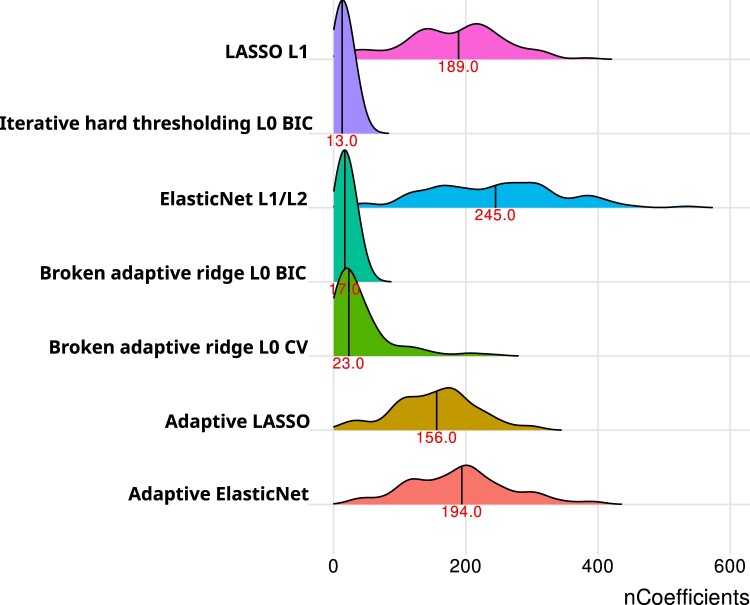
Distributions of model sizes for the 840 developed models. The vertical line and red number represent the median model size. Abbreviations: BIC = Bayesian information criteria, CV = cross validation.

Figure 4 shows the model sizes. Ridge regression is not included since it always includes all features, and its average model size is 4013 coefficients. In Figure 4 while ElasticNet is equal in discriminative performance to LASSO it does so with larger model sizes. The 2 adaptive methods have slightly lower sizes than their non-adaptive counterparts. The BAR and IHT models have by far the smallest model sizes with an average of 17 and 13 coefficients in the model, respectively. When we used CV instead of BIC to determine penalty results we found slightly higher median model size for BAR as well as a longer tail towards higher model sizes.

## Discussion

This empirical study helps researchers developing models choose whether to pick a regularization technique that maximizes discriminative performance (ie, use LASSO or ElasticNet) or whether they would be happy to achieve a slightly less discriminative model that requires fewer features and is therefore easier to understand and implement (ie, use IHT or BAR). Most researchers tend to use ridge, LASSO, or ElasticNet as regularization techniques, but our results empirically investigate the impact of regularization technique across 21 prediction problems and 5 databases. These results now provide guidance into the impact of regularization choice on discrimination, calibration, and model parsimony.

Our results show that logistic regression with the LASSO and ElasticNet regularization techniques lead to improved discrimination over the other techniques investigated. This discrimination remains consistent between the internal performance and external performance. However, the approximate L0 based techniques lead to improved parsimony and internal calibration. Internal and external calibration patterns differed, where internally IHT and BAR have better calibration but externally it was harder to see a superior technique, although LASSO and BAR tend to be slightly better calibrated. Overall, our results stand consistent with prior theoretical work, that shows L0 techniques lead to improved parsimony and calibration.^26^ One novel insight is that the improvement in calibration of the L0 techniques does not hold when models are externally validated.

If model discrimination is the only important criterion for a model developer using logistic regression, then the developer would likely want to pick either ElasticNet or LASSO regularization. These 2 regularization techniques resulted in similar AUC across the prediction problems and the AUC was higher than alternative regularization techniques investigated. ElasticNet results in more features being included in the final model compared to LASSO, with medians of 245 and 189, respectively. Therefore, if model discrimination is very important, but the developer still has some constraint on model complexity, the developer may prefer LASSO over ElasticNet. Implementing such a model with a few hundred features would not be feasible manually, but it could be implemented as part of an EHR system.

If a researcher developing models knows that implementing a model with hundreds of features would be difficult, for example if it requires manually inserting features into a form or they value understanding the model, then the researcher may prefer to use L0 based regularization such as IHT or BAR. A median of 13 and 17 features were required across the prediction task for IHT and BAR, respectively. This means these models would be simple to implement and clinicians would be able to understand the models more compared to the LASSO models that required a median of 189 features. Most developed models are not clinically implemented, and these simpler models could be an attractive choice for clinicians.^27^

While the ranks in discrimination are statistically significant and the average AUC difference is 2.8% points between best and worst algorithm, it is difficult to say what this means for a specific clinical use case. One tool to do such things is decision curve analysis where the gain to detecting one more true positive is compared to the cost of increased false positives.^28^ This requires selecting a threshold or range of thresholds which depend on the intended clinical use case or even preference of the patient. That is not possible at the large scale of comparisons done in this article.

Exploring the potential reasons behind the underperformance of adaptive LASSO and ElasticNet compared to their standard counterparts in our study, we identify several factors that might influence these findings. First, the initial coefficient estimates crucial for these methods are derived from ridge regression, which, while helpful in high-dimensional and collinear settings, introduces bias that could affect subsequent adaptive penalization. Despite utilizing a gamma value of one, as typically recommended, the possibility remains that an alternate gamma setting might yield better performance in our data context. Furthermore, the choice of using 3-fold cross-validation for determining the optimal penalization parameter was informed by prior studies focused on LASSO but could be suboptimal for adaptive methods. Future research could investigate some of these factors to improve model fitting performance with adaptive LASSO and ElasticNet in similar high-dimensional analyses.

Our study’s strengths include comparing multiple regularization techniques across 21 prediction problems and 5 databases. In addition, we investigated calibration, discrimination, and model parsimony both internally and externally. However, our study has some weaknesses. Firstly, we only investigated one target population and future work should expand this study to more target populations. Secondly, we focused on logistic regression, but the regularization techniques can be applied to different supervised learning methods and the results found for logistic regression may not generalize to other supervised learning methods. In future work it would be interesting to explore whether the results generalize to other supervised learning methods. We do not explore all possible penalties, we select penalties to investigate based on if they are commonly used in the literature and have performant implementations available suitable for our large scale comparison. Other penalties such as SLOPE or SCAD could be interesting to investigate as well in the future.^29^^,^^30^ Finally, we focused mostly on binary features and it is unknown whether the results would generalize to continuous features. However, most of the implemented clinical prediction models use binary features, such as Well’s criteria, CHA_2_DS_2_VASc, HAS-BLED, and Charlson comorbidity index.^31–34^

The literature shows that logistic regression is a common modelling technique used by researchers developing healthcare prediction models. When using big healthcare data to develop prediction models, a situation that is becoming more common, regularization is required to limit overfitting. Our large-scale empirical study can be used to guide researchers who need to select which regularization techniques to implement. If model parsimony and understanding is not important, that LASSO regularization is a good tradeoff between performance and number of features, but if model parsimony and understanding is important, then BAR often performs well while only including a manageable number of features.

## Supplementary Material

ocae109_Supplementary_Data

## Data Availability

The data underlying this article cannot be shared publicly due to the privacy of individuals whose data was used in the study. Aggregated data to support the findings of this study can be made available upon a reasonable request from the corresponding author.
