# Supplementary material for: Comparing penalization methods for linear models on large observational health data
Source: J Am Med Inform Assoc. 2024 May 20;31(7):1514–21. doi: 10.1093/jamia/ocae109 (PMC11187433; doi:10.1093/jamia/ocae109)
Supplement: ocae109_Supplementary_Data [file ocae109_supplementary_data.docx]

**SUPPLEMENTARY**


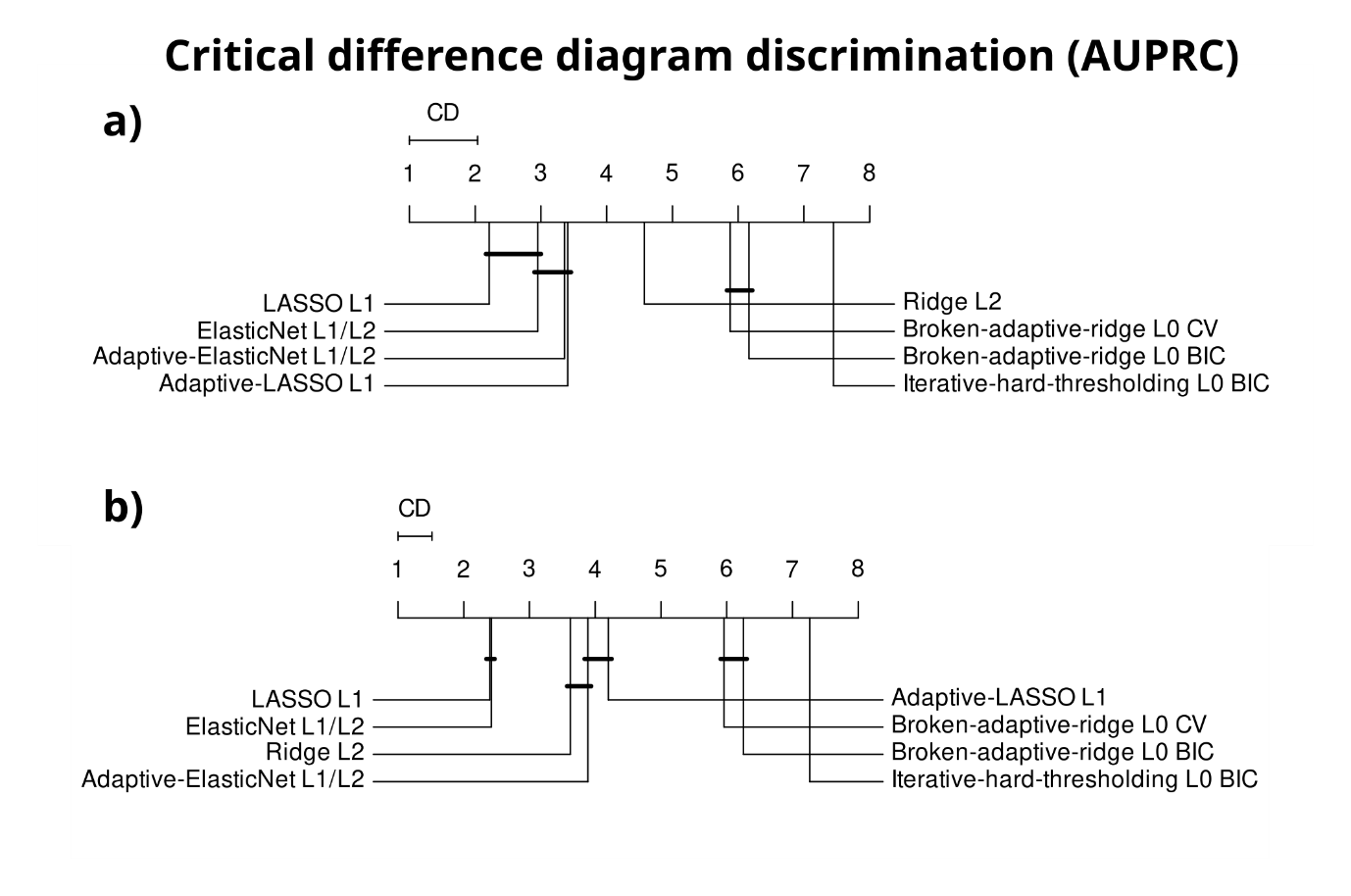


Supplementary Figure 1: a) Critical difference diagrams of internal AUPRC. b) critical difference diagram of AUPRC.

Supplementary table 1: Internal validation discrimination (AUC) ranks per outcome.

| outcomes | LASSO L1 | ElasticNet L1/L2 | Ridge L2 | Adaptive-ElasticNet L1/L2 | Adaptive-LASSO L1 | BAR L0 BIC | BAR L0 CV | IHT L0 BIC |
| --- | --- | --- | --- | --- | --- | --- | --- | --- |
| acute liver injury | 2 | 2.25 | 2.25 | 4.75 | 4.75 | 7.25 | 5.75 | 7 |
| acute myocardial infarction | 1.6 | 1.4 | 5.4 | 4.2 | 3.6 | 7.2 | 5.4 | 7.2 |
| alopecia | 2.8 | 2.8 | 1 | 4.4 | 4.4 | 6.4 | 6.7 | 7.5 |
| constipation | 2 | 2.4 | 1.6 | 4.4 | 4.6 | 7 | 6.2 | 7.8 |
| decreased libido | 2.25 | 1.25 | 2.75 | 4.25 | 4.5 | 7 | 6.25 | 7.75 |
| delirium | 2.6 | 2 | 1.6 | 4.2 | 4.8 | 6.6 | 6.8 | 7.4 |
| diarrhea | 2.6 | 2.4 | 1 | 4.8 | 4.6 | 7.4 | 5.8 | 7.4 |
| fracture | 1.8 | 1.6 | 4.2 | 3.8 | 3.8 | 6.2 | 6.8 | 7.8 |
| gastrointestinal hemorrhage | 1.8 | 2 | 2.6 | 3.8 | 4.8 | 6.8 | 6.6 | 7.6 |
| hypoatremia | 1.4 | 1.8 | 3.6 | 4 | 5 | 7.2 | 5.4 | 7.6 |
| hypotension | 1.8 | 1.6 | 3 | 4 | 4.6 | 7 | 6.6 | 7.4 |
| hypothyroidism | 1.8 | 1.6 | 5 | 4.6 | 4.2 | 5.8 | 5.4 | 7.6 |
| insomnia | 1.4 | 1.6 | 3.6 | 4.6 | 4.8 | 7.4 | 5 | 7.6 |
| ischemic stroke | 1.8 | 1.6 | 3.2 | 4.4 | 4 | 7.2 | 6.4 | 7.4 |
| nausea | 2.6 | 2 | 1.4 | 4.2 | 5 | 7.4 | 5.8 | 7.6 |
| open-angle glaucoma | 1.2 | 2.8 | 7 | 5.6 | 2.8 | 4.6 | 5.4 | 6.6 |
| seizure | 2.6 | 1.6 | 1.8 | 4.4 | 5 | 7 | 5.8 | 7.8 |
| suicide and ideation | 1.8 | 1.2 | 4.4 | 4 | 3.8 | 7.6 | 6 | 7.2 |
| tinnitus | 2 | 1.4 | 3.8 | 4.2 | 3.6 | 7 | 6.2 | 7.8 |
| ventricular arrhythmia | 1.6 | 1.4 | 3 | 5 | 4.8 | 6.8 | 5.6 | 7.8 |
| vertigo | 1.8 | 1.2 | 4.2 | 4.4 | 3.4 | 7.6 | 6 | 7.4 |
| Average rank | 1.96 | 1.80 | 3.16 | 4.38 | 4.33 | 6.88 | 6.00 | 7.49 |

BAR: Broken adaptive ridge, IHT: Iterative hard thresholding, BIC: Bayesian information criteria, CV: Cross validation

Supplementary table 2: External validation discrimination (AUC) ranks per outcome.

| outcomes | LASSO L1 | ElasticNet L1/L2 | Ridge L2 | Adaptive-ElasticNet L1/L2 | Adaptive-LASSO L1 | BAR L0 BIC | BAR L0 CV | IHT L0 BIC |
| --- | --- | --- | --- | --- | --- | --- | --- | --- |
| acute liver injury | 2.4 | 1.6 | 2.0 | 5.5 | 5.1 | 6.4 | 5.8 | 7.0 |
| acute myocardial infarction | 1.4 | 2.4 | 6.5 | 4.4 | 3.4 | 6.6 | 5.1 | 6.4 |
| alopecia | 2.7 | 2.0 | 1.4 | 4.4 | 5.0 | 7.3 | 6.3 | 7.1 |
| constipation | 3.3 | 2.2 | 1.1 | 4.3 | 4.5 | 7.4 | 6.3 | 7.0 |
| decreased libido | 3.2 | 2.0 | 1.7 | 4.3 | 4.4 | 6.9 | 6.3 | 7.3 |
| delirium | 2.8 | 1.9 | 2.3 | 3.9 | 4.6 | 6.9 | 6.6 | 7.2 |
| diarrhea | 3.0 | 2.0 | 1.0 | 4.9 | 4.8 | 7.7 | 5.4 | 7.4 |
| fracture | 2.3 | 3.2 | 4.7 | 3.8 | 3.4 | 5.1 | 6.2 | 7.6 |
| gastrointestinal hemorrhage | 1.8 | 2.0 | 3.2 | 3.8 | 4.7 | 7.0 | 6.4 | 7.3 |
| hypoatremia | 1.4 | 1.8 | 4.9 | 5.4 | 5.1 | 6.4 | 4.8 | 6.4 |
| hypotension | 2.1 | 1.8 | 2.6 | 4.1 | 4.8 | 7.2 | 6.4 | 7.2 |
| hypothyroidism | 1.7 | 3.0 | 6.7 | 5.6 | 5.1 | 3.2 | 5.2 | 5.7 |
| insomnia | 1.9 | 1.7 | 3.4 | 4.4 | 4.5 | 7.4 | 5.4 | 7.6 |
| ischemic stroke | 2.7 | 3.0 | 3.6 | 3.9 | 3.7 | 6.1 | 5.7 | 7.5 |
| nausea | 2.7 | 2.1 | 1.3 | 4.5 | 4.7 | 7.3 | 6.2 | 7.4 |
| open-angle glaucoma | 1.8 | 2.9 | 6.5 | 5.2 | 3.6 | 4.4 | 5.6 | 6.2 |
| seizure | 2.9 | 1.9 | 1.7 | 4.1 | 4.7 | 7.2 | 6.2 | 7.4 |
| suicide and ideation | 2.4 | 2.3 | 5.2 | 3.9 | 4.1 | 6.7 | 4.7 | 6.9 |
| tinnitus | 2.4 | 1.8 | 2.6 | 4.3 | 4.6 | 7.1 | 5.9 | 7.6 |
| ventricular arrhythmia | 1.5 | 1.9 | 3.5 | 4.8 | 5.0 | 6.2 | 6.2 | 7.0 |
| vertigo | 2.3 | 1.5 | 3.4 | 4.3 | 3.7 | 7.5 | 6.2 | 7.4 |
| Average rank | 2.30 | 2.12 | 3.27 | 4.45 | 4.43 | 6.56 | 5.82 | 7.05 |

BAR: Broken adaptive ridge, IHT: Iterative hard thresholding, BIC: Bayesian information criteria, CV: Cross validation

Software used:

To extract features FeatureExtraction (ver 3.2.0) was used^[[1]](#footnote-1)^. This is a package that allows extracting standard sets of features from databases in the CDM. It does so by translating R code into sql queries that extract the data from the database.

For model development we use PatientLevelPrediction (ver 5.4.5). This is an R package that provides a common interface to develop models using best practices on data in the CDM. In most cases it calls other packages which have the model fitting algorithm itself. In our case, LASSO and Ridge use the Cyclops R package^[[2]](#footnote-2)^ (version 3.2.0) for model fitting[5]. ElasticNet, Adaptive LASSO and Adaptive ElasticNet uses glmnet^[[3]](#footnote-3)^ (version 4.1.4) while IHT (version 1.0.2) and BAR (version 1.0.0) each use their own R package^[[4]](#footnote-4),^^[[5]](#footnote-5)^ which use Cyclops under the hood.

1. <https://github.com/OHDSI/FeatureExtraction> [↑](#footnote-ref-1)
2. <https://github.com/OHDSI/Cyclops/> [↑](#footnote-ref-2)
3. <https://glmnet.stanford.edu/> [↑](#footnote-ref-3)
4. <https://github.com/OHDSI/IterativeHardThresholding/> [↑](#footnote-ref-4)
5. <https://github.com/OHDSI/BrokenAdaptiveRidge/> [↑](#footnote-ref-5)
